# Prevalence of Diabetes and Pre-Diabetes and Associated Risk Factors among Tuberculosis Patients in India

**DOI:** 10.1371/journal.pone.0041367

**Published:** 2012-07-26

**Authors:** Vijay Viswanathan, Satyavani Kumpatla, Vigneswari Aravindalochanan, Rajeswari Rajan, C. Chinnasamy, Rajan Srinivasan, Jerard Maria Selvam, Anil Kapur

**Affiliations:** 1 Diabetology, M.V. Hospital for Diabetes and Prof. M. Viswanathan Diabetes Research Centre (WHO Collaborating Centre for Research, Education and Training in Diabetes), Chennai, Tamil Nadu, India; 2 Health Department, Directorate of Medical Services, Chennai, Tamil Nadu, India; 3 Health Department, WHO–RNTCP Technical Assistance Project, Chennai, Tamil Nadu, India; 4 Tamil Nadu State Health Department, Tamil Nadu Health Systems Project, Chennai, Tamil Nadu, India; 5 World Diabetes Foundation, Brogårdsvej, Gentofte, Denmark; McGill University, Canada

## Abstract

**Background:**

Diabetes mellitus (DM) is recognised as an important risk factor to tuberculosis (TB). India has high TB burden, along with rising DM prevalence. There are inadequate data on prevalence of DM and pre-diabetes among TB cases in India. Aim was to determine diabetes prevalence among a cohort of TB cases registered under Revised National Tuberculosis Control Program in selected TB units in Tamil Nadu, India, and assess pattern of diabetes management amongst known cases.

**Methods:**

827 among the eligible patients (n = 904) underwent HbA1c and anthropometric measurements. OGTT was done for patients without previous history of DM and diagnosis was based on WHO criteria. Details of current treatment regimen of TB and DM and DM complications, if any, were recorded. A pretested questionnaire was used to collect information on sociodemographics, habitual risk factors, and type of TB.

**Findings:**

DM prevalence was 25.3% (95% CI 22.6–28.5) and that of pre-diabetes 24.5% (95% CI 20.4–27.6). Risk factors associated with DM among TB patients were age (31–35, 36–40, 41–45, 46–50, >50 years vs <30 years) [OR (95% CI) 6.75 (2.36–19.3); 10.46 (3.95–27.7); 18.63 (6.58–52.7); 11.05 (4.31–28.4); 24.7 (9.73–62.7) (p<0.001)], positive family history of DM [3.08 (1.73–5.5) (p<0.001)], sedentary occupation [1.69 (1.10–2.59) (p = 0.016)], and BMI (18.5–22.9, 23–24.9 and ≥25 kg/m^2^ vs <18.5 kg/m^2^) [2.03 (1.32–3.12) (p = 0.001); 0.87 (0.31–2.43) (p = 0.78); 1.44 (0.54–3.8) (p = 0.47)]; for pre-diabetes, risk factors were age (36–40, 41–45, 46–50, >50 years vs <30 years) [2.24 (1.1–4.55) (p = 0.026); 6.96 (3.3–14.7); 3.44 (1.83–6.48); 4.3 (2.25–8.2) (p<0.001)], waist circumference [<90 vs. ≥90 cm (men), <80 vs. ≥80 cm (women)] [3.05 (1.35–6.9) (p = 0.007)], smoking [1.92 (1.12–3.28) (p = 0.017)] and monthly income (5000–10,000 INR vs <5000 INR) [0.59 (0.37–0.94) (p = 0.026)]. DM risk was higher among pulmonary TB [3.06 (1.69–5.52) (p<0.001)], especially sputum positive, than non-pulmonary TB.

**Interpretation:**

Nearly 50% of TB patients had either diabetes or pre-diabetes.

## Introduction

The global burden of diabetes mellitus (DM) and tuberculosis (TB) is huge. Nearly one-third of world’s population is infected with *Mycobacterium tuberculosis* and about 10% of them are at risk of developing active form of the disease in their lifetime depending upon the interaction of the epidemiological triad [1], [2]. Available reports suggest that 95% of patients with TB live in the low- and middle-income countries and more than 70% of patients with DM also live in the same countries, especially in South East Asia.

India has the largest number of TB cases estimated globally to be 2 million per annum and accounts for more than 60 million people with type 2 diabetes [3]. Globally 1.1–1.7 million people die from TB every year and a substantial number of these deaths occur in India [4]. Significant improvements in case detection and control of TB disease [5] have been noted suggesting that India may be on track to achieve the STOPTB goal of halving the prevalence and deaths due to TB by 2015, compared to 1990 level. However, the recognised challenges in this breakthrough were MDR TB, HIVTB co-infection, and so on [6]. Another important challenge is the growing body of evidence suggesting co-existing diabetes as a risk factor for new as well as reactivation of old cases of TB [7].

Many studies reported that subjects with diabetes were at three-fold higher risk of developing TB. In addition, studies that screened for DM among TB patients reported a wide range of DM prevalence among TB patients, ranging from 1.9% to as high as 35% [8], [9]. A secondary data analysis on Indian population revealed that with an estimated 21 million adults with DM and 900,000 incident pulmonary tuberculosis (PTB) cases in 2000, 14.8% of the existing TB burden could be attributed to diabetes [10]. A recent nationwide study conducted in 2011 reported the prevalence rates of diabetes and pre-diabetes to be 10.4% and 8.3%, respectively, among the general population of Tamil Nadu, South India [11].

Therefore, it is necessary to consider the increasing prevalence of diabetes since diabetes is associated with an increased risk of TB treatment failure and death during tuberculosis treatment, as well as an increased risk of relapse. A study from Jaipur, Rajasthan, which followed up TB cases taking Category I and II treatment for 2 years, reported diabetes as one of the associated factors for relapse of TB cases [12]. Similar findings were also reported in a study conducted amongst Chinese population [13].

Screening for DM in TB patients could improve DM case detection and early treatment and indirectly lead to better TB-specific treatment outcomes [14]. Many research questions regarding association between diabetes and TB remain unanswered because of lack of well-designed studies [15]. This study was planned to determine the prevalence of diabetes and pre-diabetes amongst a cohort of TB patients registered in selected Tuberculosis Units (TUs) of Revised National Tuberculosis Control Program (RNTCP) in Tamil Nadu, India, and understand the pattern of diabetes management availed by the known diabetes cases.

## Methods

### Ethics Statement

Ethics Committee of Prof. M. Viswanathan Diabetes Research Centre approved the study and written consent was obtained from all study subjects. Tamil Nadu state RNTCP also gave permission to conduct the study.

### Study Population

**Figure 1:**
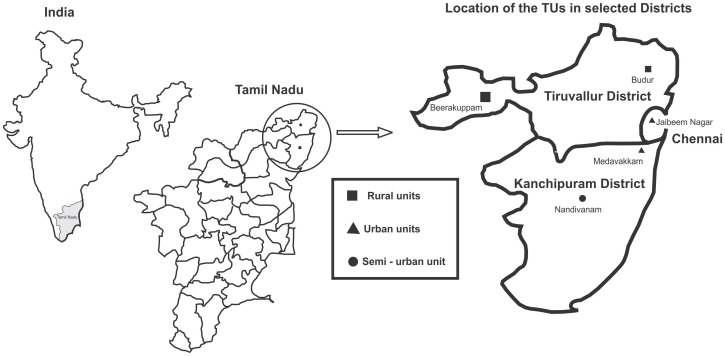
Location of selected Tuberculosis Units in three districts of Tamil Nadu, India.

The study population was selected from Tamil Nadu state in South India. Five TUs – two from urban (Jaibeem Nagar and Medavakkam), two from rural (Budur and Beerakuppam), and one from semi-urban (Nandivaram) areas of Chennai, Thiruvallur, and Kanchipuram districts, respectively, of Tamil Nadu were randomly selected for this study [Figure 1]. Each TB unit has a defined catchment area of 500,000 population as mentioned under RNTCP. All these TB units were stratified depending upon their location to urban, semi-urban and rural areas, out of which 2 urban, 2 rural and 1 semi-urban were randomly selected from these three different strata. All those who registered for Directly Observed Treatment, Short Course (DOTS) in these TUs during the first quarter of 2011 i.e., from January–March 2011 and meeting the selection criteria age ≥18 years, PTB cases confirmed with sputum smear for acid fast bacilli and X-ray, and extrapulmonary cases confirmed with culture of specimen from the site and or histological evidence were included. Diagnosis of TB was based on standard diagnostic criteria of RNTCP [16]. Subjects with type 1 diabetes were excluded from the study.

**Figure 2:**
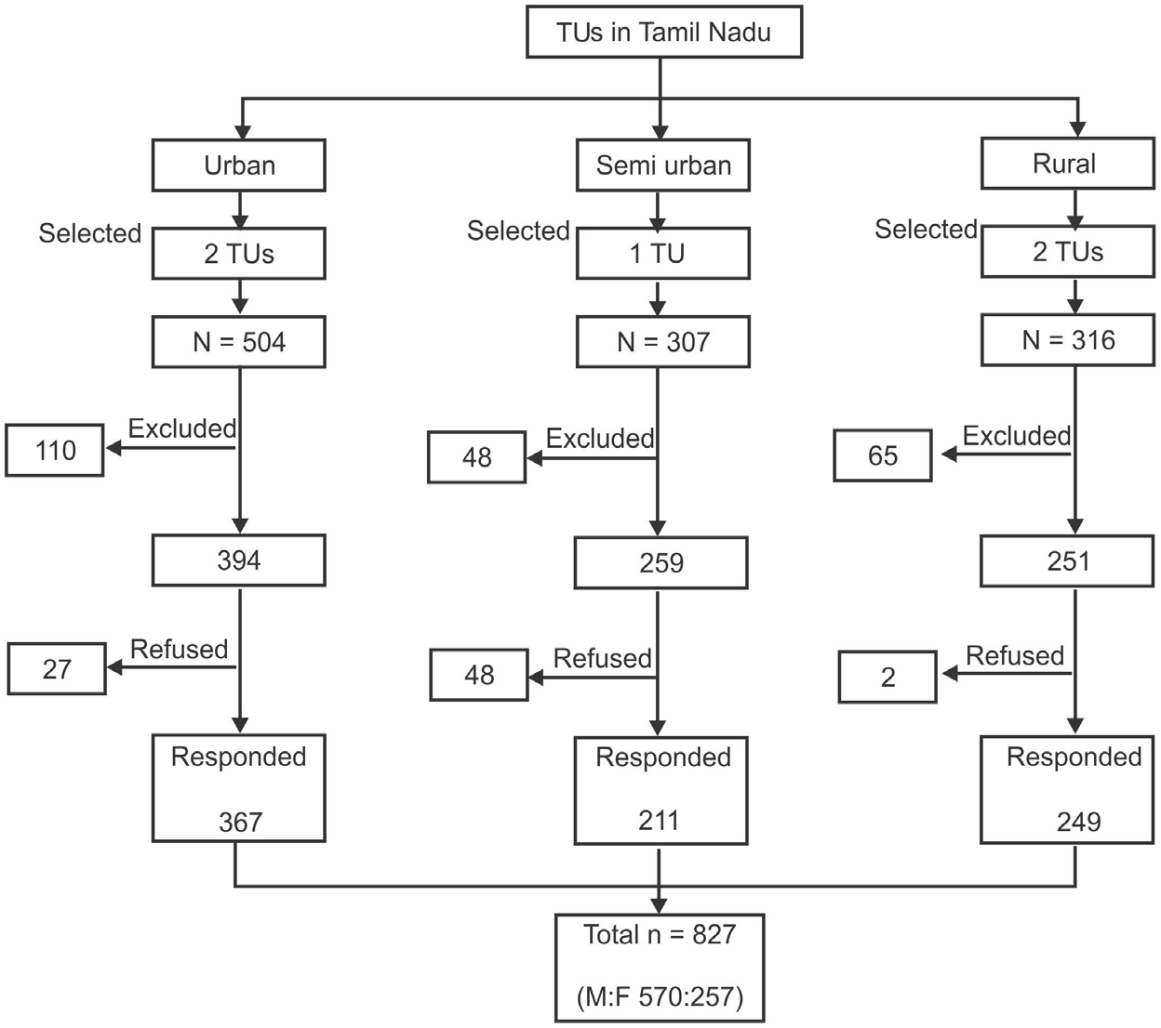
Flowchart showing the details of selection of study sites and study subjects.

After identification of the sampling frame, cluster random sampling technique was used to recruit the study subjects. A sample size of 800 was required, assuming the prevalence of diabetes as 20% amongst the TB subjects, with 95% confidence interval, precision ±5%, non-response rate of 10% and with the inclusion of design effect of 2. The assumption of prevalence of 20% (for 50.8 million people with diabetes in 2010) was based on the prevalence of DM among TB patients estimated by Stevenson et al. as 18.4% for 20 million people with diabetes in India in 2000 [10]. Figure 2 shows the flowchart of selection of study sites and study subjects. A total of 1127 patients were registered for TB treatment in the above five TUs of which, 904 were eligible for the study. A total of 827 (M:F 510∶257) patients underwent diabetes screening, out of which 695 were newly registered TB cases.

A trained investigator recorded anthropometric measurements viz., height, weight, and waist circumference by standard procedure. BMI (kg/m^2^) was calculated. Two blood pressure measurements were taken using sphygmomanometer with the subject in sitting posture, and average of the two readings was recorded.

Fasting venous blood sample was taken and all patients underwent a standard 2 hr 75 g Oral Glucose Tolerance Test. Diagnosis of diabetes was based on previous history of diabetes or on WHO criteria for the classification of glucose tolerance [17]. Fasting and postprandial samples were collected from known cases of diabetes. Plasma glucose was estimated by glucose oxidase peroxidase method. Glycosylated haemoglobin (HbA1c) was measured by HPLC method using BIORAD variant turbo equipment. Renal parameters such as urea and creatinine were estimated by standard methods. Total cholesterol was estimated by enzymatic procedure using fasting serum sample.

A pretested questionnaire was administered to collect information regarding sociodemographics and habitual risk factors viz., smoking, alcohol consumption, and other forms of tobacco use; family history of TB and DM, educational and occupational status, and monthly income. Type of TB, status of TB treatment, and category of treatment were also recorded. Patients already diagnosed with diabetes were interviewed to elicit and record information on duration of diabetes, complications if any, the place where treatment was being undertaken, type of treatment, and adherence to treatment.

### Statistical Analysis

SPSS version 16.0 was used for statistical analysis. Prevalences are reported with 95% confidence intervals calculated considering the design effect. Mean and standard deviation for continuous variables and proportions for categorical variables are reported. Independent sample ‘t’ test and ANOVA were used to test continuous variables and Chi-square test was used to compare categorical variables. Multinomial logistic regression analysis was performed to identify the factors associated with diabetes and pre-diabetes – the dependent variable being either diabetes or pre-diabetes and the independent variables were age [(years) (categorised in 5 units; <30 (reference), 31–35, 36–40, 41–45, 46–50, >50)], sex, occupation (sedentary vs non-sedentary), locality (urban vs rural), monthly income [(INR) (categorised as <5000, 5000–10,000 and >10,000)], BMI [(kg/m^2^) (categorised as <18.5 (reference), 18–22.9, 23.0–24.9, ≥25.0)]), waist circumference [<90 vs. ≥90 cm (men), <80 vs. ≥80 cm (women)], type of TB (pulmonary vs extrapulmonary), family history of DM, smoking, alcohol consumption, and tobacco use. The model fit information was significant for multinomial logistic regression analysis (χ^2^ = 221.1, p<0.001) and also the fit was good (χ^2^ = 1048.7, p = 0.129). A P value of <0.05 was considered statistically significant.

## Results

The details on the total number of eligible participants in each TU and those who have given written consent to undergo screening of DM are illustrated in a flow chart [Figure 2]. The recorded response rate was 91.5%.

### General Characteristics of the Study Subjects

The mean age of the study subjects was 41.1±14.4 years. 151 (18.3%) subjects were aged less than 30 years, 78 (9.4%) aged between 31–35 years, 114 (13.8%) of them were aged between 36–40 years, 89 (10.8%) subjects were aged between 41–45 years, 181 (21.9%) between 46–50 years, and 214 (25.9%) of them were aged >50 years. Mean BMI of the TB subjects was 18.5±4. 473 (57.2%) of them had BMI (kg/m^2^) <18.5 (underweight), 259 (31.3%) had BMI 18.5–22.9 (normal BMI), 37 (4.5%) had BMI 23.0–24.9 (overweight), and 58 (7%) had BMI ≥25 (obese).

The gender-wise demographic and anthropometric information is detailed in Table 1. Women were comparatively younger than men (35.6±14 vs. 43.6±13.9 years; p<0.001). Mean BMI of women was significantly higher than that of their male counterparts (19.7±5 vs. 18±3.3 kg/m^2^; p<0.001). There was no significant difference in the waist circumference and systolic blood pressure whereas diastolic blood pressure was higher in men compared to women (75.9±10.5 vs. 73.1±9 mmHg; p<0.001). Gender difference was not noted in the dwelling areas, i.e., urban vs rural. Gender difference was seen in educational status, and majority of men were employed compared to women (67% vs. 24.9%; p<0.001). Monthly income was similar between men and women (p = 0.380). About 2% of men gave history of smoking or consuming tobacco in other form and consuming alcohol. The proportion of subjects with positive family history of either TB or DM was also similar between men and women (p = 0.623 and p = 0.648).

**Table 1:** Demographic and anthropometric details of the study subjects.

Characteristics	Total (n = 827)	Men (n = 570)	Women (n = 257)	P value (Men vs. Women)
Values are mean ±SD				
**Age** (years)	41.1±14.4	43.6±13.9	35.6±14	<0.001
BMI (kg/m^2^)	18.5±4	18±3.3	19.7±5	<0.001
Waist Circumference (cm)	72.2±11.9	72.1±11.1	72.5±13.7	0.931
**Blood Pressure (mm/Hg)**				
Systolic	112.3±13.9	111.3±13.6	109.8±14.2	0.147
Diastolic	75±10.1	75.9±10.5	73.1±9	<0.001
Values are n (%)				
**Location**				
Urban	578(69.9)	396(69.5)	182 (70.8)	0.810
Rural	249(30.1)	174(30.5)	75 (29.2)	
**Educational status**				
Literate	626(75.7)	446(78.2)	180(70)	0.014
Illiterate	201(24.3)	124 (21.8)	77(30)	
**Occupation**				
Employed	446(53.9)	382 (67)	64(24.9)	<0.001
Unemployed	191(23.1)	156(27.4)	35(13.6)	
Retired	21(2.5)	19(3.3)	2(0.8)	
Housewife	142(17.2)	–	142(55.3)	
Student	27(3.3)	13 (2.3)	14(5.4)	
**Monthly Income (INR)**				
<5000	616(74.5)	419(73.5)	197(76.6)	
5000–10000	162(19.6)	113(19.8)	49(19.1)	0.380
>10000	49(5.9)	38(6.7)	11(4.3)	
**Smoking**				
Regular/heavy	15 (1.8)	15 (2.6)	–	
Occasional	11 (1.3)	11 (1.9)	–	
Ex-Habits	292 (35.3)	292 (51.2)	–	
**Alcohol consumption**				
Regular/heavy	13 (1.6)	13(2.3)	–	
Occasional	14 (1.7)	14(2.5)	–	
Ex-Habit	322 (38.9)	322(56.5)	–	
**Tobacco chewing**				
Regular/heavy	14(1.7)	13(2.3)	1(0.4)	
Occasional	9(1.1)	8 (1.4)	1(0.4)	0.001
Ex-Habit	74(8.9)	63(11)	11(4.3)	
**Snuff use**				
Regular/heavy	1(0.1)	–	1(0.4)	
Occasional	2(0.2)	2(0.4)	–	0.431
Ex-Habit	13(1.6)	10(1.8)	3(1.2)	
**Family History of DM**	92(11.1)	61(10.7)	31(12.1)	0.648
**Family History of TB**	125(15.1)	89(15.6)	36(14.0)	0.623

### Prevalence of Diabetes and Pre-diabetes among TB Patients

Out of 827 patients, 209 (25.3%) [95% CI 22.6–28.5] had diabetes and prevalence of diabetes was significantly higher in men compared to women [27.5 (95% CI 22.8–31.2) vs 20.2% (95% CI 15.4–26.2); p = 0.031). Diabetes was newly detected in 9.3% (n = 77) of TB patients and 15.96% (n = 132) were known cases of diabetes. The prevalence of pre-diabetes was 24.5% (95% CI 20.4–27.6). Subjects were undiagnosed previously and the screening process identified that they had pre-diabetes. The prevalence of isolated Impaired Fasting Glucose (IFG) was 2.7% (95% CI 1.4–3.9) and isolated Impaired Glucose Tolerance (IGT) was 16.3% (95% CI 13.2–18.1) and both IFG and IGT were present in 5.6% (95% CI 4.0–7.5) of the subjects. There was no significant difference noted between men and women in the prevalence of different categories of pre-diabetes [Table 2].

**Table 2:** Prevalence of diabetes and pre diabetes in South Indian TB patients.

	Diabetes n (%) [95%confidence interval (CI)]	Pre diabetes n (%) [95% CI]			
		Total	IFG	IGT	IFG & IGT
Total (n = 827)	209 (25.3) [22.6–28.5]	203 (24.5) [20.4–27.6]	22 (2.7) [1.4–3.9]	135 (16.3) [13.2–18.1]	46(5.6) [4.0–7.5]
Men (n = 570)	157 (27.5) [22.8–31.2]	143 (25.1) [21.3–28.6]	16 (2.8) [1.6–3.1]	96 (16.8) [13.8–19.3]	30 (5.3) [3.4–7.5]
Women (n = 257)	52 (20.2) [15.4–26.2]	60 (23.3) [18.2–28.4]	6 (2.3) [1.3–4.4]	39 (15.2) [10.2–19.6]	16 (6.2) [3.0–9.7]

**Men Vs Women:**

Diabetes: χ^2^ = 4.633, p = 0.031.

Pre-diabetes: χ^2^ = 0.204, p = 0.652.

IFG: χ^2^ = 0.025, p = 0.875.

IGT: χ^2^ = 0.249, p = 0.618.

IFG & IGT: χ^2^ = 0.156, p = 0.693.

### Characteristics of TB Patients as per Stages of Glucose Intolerance

Table 3 shows the comparison of the study characteristics among patients with normoglycemia, pre-diabetes, and diabetes. TB patients with diabetes were older than the subjects with pre-diabetes and normoglycaemia (49.3±11.5 vs. 43.9±13.9 vs. 35.6±13.5 years, p<0.001). Mean BMI of patients with diabetes was higher when compared to patients with pre-diabetes and normoglycaemia (p = 0.03). Mean waist circumference of men in the three groups differed significantly (p<0.001) whereas this was not observed among women (p = 0.485). TB patients with diabetes had a higher systolic blood pressure in comparison with patients with normoglycemia and pre-diabetes (p = 0.001). Diastolic blood pressure was similar between the groups (p = 0.144). As expected, fasting and 2 hr plasma glucose levels and HbA1c were statistically significant between the groups (p<0.001). Urea and serum creatinine levels were similar whereas patients with diabetes had higher cholesterol levels (p<0.001).

**Table 3:** Comparison of study characteristics among TB patients with normoglycemia, pre-diabetes and diabetes.

	Non-DM	Pre-diabetes		Diabetes		P value		
N, M:F	415, 270∶145	203, 143∶60		209, 157∶52				
	Values are Mean ± SD							
**Age (years)**	35.6±13.5	43.9±13.9		49.3±11.5		<0.001		
**BMI (kg/m^2^)**	18.4±3.7	18.1±4.4		19.3±4.11		0.03		
**Waist circumference (cm)**								
Men	70.27±10.5	71.4±9.5		75.7±12.9		<0.001		
Women	72.2±11.8	71.5±16.6		74.4±14.8		0.485		
**Blood Pressure (mm Hg)**								
Systolic	111±13	111±14		115±14		0.001		
Diastolic	75±11	74±9		76±10		0.144		
**Plasma glucose (mmol/L)**								
Fasting	5.1±0.5	5.72±0.6		9.1±4.2		<0.001		
2 hr	6.3±0.99	8.72±1.2		15.2±5.7		<0.001		
**HbA1c%**	5.8±0.03	6.1±0.6		8.9±2.8		<0.001		
**Total Cholesterol (mmol/L)**	4.2±0.8	4.32±0.9		4.6±1.1		<0.001		
**Urea (mmol/L)**	3.76±1.1	3.89±1.6		3.86±1.4		0.418		
**Creatinine (µmol/L)**	68.1±12.4	73.4±52.2		69.8±15.0		0.092		
	Values are n (%)							
**Type of TB (n = 695)**	**n = 351**		**n = 172**		**n = 172**			
New smear positive cases	149 (42.5)		87 (50.6)		96 (55.8)		<0.001	
New smear negative cases	104 (29.6)		57 (33.1)		54 (31.4)			
New extra pulmonary TB cases	98 (27.9)		28 (16.3)		22 (12.8)			
**Relapse**	24 (5.8)		13 (6.4)		18 (8.6)			
**Treatment Default**	18 (4.3)		14 (6.9)		16 (7.7)			
**Failure**	6 (1.4)		1 (0.5)		–			
**Transferred**	1 (0.2)		–		–			
**Others**	15 (3.6)		3 (1.5)		3 (1.4)			
**Treatment category**								
Category-I	352 (84.8)		172 (84.7)		172 (82.3)		0.656	
Category-II	63 (15.2)		31 (15.3)		37 (17.7)			

### Tuberculosis Profile of the Study Patients

Among the newly registered TB cases (695), 172 patients had diabetes and another 172 had pre-diabetes. A higher proportion of patients with TB DM were smear positive (55.8%) compared to pre-diabetes (50.6%) or those with normoglycaemia (42.5%). Extrapulmonary TB on the other hand was more common among TB patients with normoglycaemia (27.9%) compared to patients with pre-diabetes (16.3%) and diabetes (12.8%) as shown in Table 3. More than 80% in all the three groups were receiving Category-I treatment. A small proportion of patients were categorised as relapse, treatment defaulters, and failure cases in all categories of glucose intolerance and differences were not statistically significant [NonDM vs PreDM vs DM; Relapsed cases (5.8 vs 6.4 vs 8.6%), treatment defaulters (4.3 vs 6.9 vs 7.7%), and failure cases (1.4 vs 0.5 vs 0%)]. The median period of duration of TB was 22 days (1–45 days) at the time of screening for DM.

### Risk Factors Associated with Diabetes and Pre-diabetes among TB Patients

Multinomial logistic regression analysis showed that increasing age, BMI category 18.5–22.9 kg/m^2^, positive family history of diabetes, sedentary occupation and presence of PTB were significantly associated with diabetes among TB patients. Age category >50 years had a great influence with odds ratio of 24.7 (95% CI 9.73–62.7, p<0.001), followed by positive family history of diabetes with OR 3.08 (95% CI 1.73–5.5). PTB compared to non-PTB was associated with a higher risk of diabetes with an odds ratio of 3.06 (95% CI 1.69–5.52, p<0.001), [Table 4]. Age, waist circumference, smoking habit, and monthly income of 5000–10,000 INR were significantly associated with pre-diabetes among TB patients. The odds ratio was highest for the age group 41–45 years (OR 6.96, 95% CI 3.3–14.7, p<0.001) and for waist circumference (OR 3.05; 95% CI 1.35–6.9) in pre-diabetes [Table 5].

**Table 4:** Results of multinomial logistic regression analysis (Diabetes vs. Non-diabetes; dependent variable).

Significant variables	β	SEβ	Odds Ratio (95% Confidence Interval)	P value
**Age (years)**				
<30 (reference)	–	–	1.0	–
31–35	1.91	0.54	6.75 (2.36–19.32)	<0.001
36–40	2.35	0.49	10.46 (3.95–27.7)	<0.001
41–45	2.93	0.53	18.63 (6.58–52.7)	<0.001
46–50	2.40	0.48	11.05 (4.31–28.4)	<0.001
>50	3.21	0.48	24.7 (9.73–62.7)	<0.001
**Pulmonary tuberculosis**	1.12	0.30	3.06 (1.69–5.52)	<0.001
**Positive family history of DM**	1.13	0.29	3.08 (1.73–5.5)	<0.001
**Sedentary occupation**	0.52	0.22	1.69 (1.10–2.59)	0.016
**Waist circumference** (≥90 cm for men; ≥80 cm for women)	0.79	0.42	2.22 (0.97–5.1)	0.058
**BMI (kg/m^2^)**				
<18.5 (reference)	–	–	–	–
18.5–22.9	0.71	0.22	2.03 (1.32–3.12)	0.001
23–24.9	0.14	0.53	0.87 (0.31–2.43)	0.78
≥25.0	0.36	0.50	1.44 (0.54–3.8)	0.47

**Nonsignificant:** Gender, Locality, Smoking, Alcohol consumption, Tobacco use, Monthly income.

**Table 5:** Results of multinomial logistic regression analysis (Pre-diabetes vs. Non-diabetes; dependent variable).

Significant variables	β	SEβ	Odds Ratio (95% Confidence Interval)	P value
**Age (years)**				
<30 (reference)	–	–	1.0	–
31–35	0.67	0.40	1.96 (0.89–4.27)	0.092
36–40	0.81	0.36	2.24 (1.1–4.55)	0.026
41–45	1.94	0.38	6.96 (3.3–14.7)	<0.001
46–50	1.24	0.32	3.44 (1.83–6.48)	<0.001
>50	1.46	0.33	4.3 (2.25–8.2)	<0.001
**Waist circumference** (≥90 cm for men;≥80 cm for women)	1.11	0.42	3.05 (1.35–6.9)	0.007
**Smoking habit**	0.65	0.27	1.92 (1.12–3.28)	0.017
**Monthly income (INR)**				
<5000 (reference)	–	–	–	–
5000–10,000	0.53	0.24	0.59 (0.37–0.94)	0.026
>10,000	0.32	0.33	0.72 (0.38–1.4)	0.328

**Nonsignificant variables:** Gender, Locality, Alcohol consumption, Tobacco use, BMI, Type of TB, Occupation.

### Treatment Pattern and Presence of Complications in Previously Diagnosed Cases of DM

Out of 209 DM subjects, 71 (34%) had HbA1c <7%, 25 (12%) had HbA1c 7.0–7.9%, 29 (13.9%) had HbA1c 8.0–8.9%, 15 (7.2%) had HbA1c 9.0–9.9%, and 69 (33%) had HbA1c ≥10.0%. Moreover, there was no significant difference in the proportion of new and relapsed cases between the HbA1c categories (p = 0.634).

The median period of duration of diabetes among known cases was 2.3 years (0.5–24 years). Among the known cases of DM, 92.4% (n = 122) subjects reported regular compliance to treatment for diabetes, the remaining reported intermittent treatment or irregular compliance to diabetes treatment. Among those who were on regular treatment, 84 (68.9%) were on oral hypoglycemic agents, 29 (23.8%) were only on insulin, and nine (7.4%) were taking both oral hypoglycemic agents and insulin. About 59% (n = 72) were undergoing treatment in government health centres and 41% (n = 50) were undertaking treatment in private healthcare settings. Complications and comorbid conditions reported amongst subjects with pre-existing diabetes were nine cases (6.8%) of cardiovascular disorders, 12 cases (9.1%) of hypertension, three cases with (2.3%) foot ulcers, and one case (0.7%) of retinopathy.

## Discussion

The association between DM and TB is well documented and there is substantial evidence to support the fact that diabetes is an important risk factor for TB [9]. Conversely, it is also possible that TB can induce glucose intolerance and also deteriorate glycemic control in subjects with diabetes [18]. Previously there were no data from this area on the prevalence of both diagnosed and undiagnosed DM among TB patients. The present study showed that prevalence rates of DM and pre-diabetes were 25.3% and 24.5% respectively among TB patients registered under RNTCP in South India. Diabetes was more prevalent among men than women (27.5% vs. 20.2%) while there was no gender difference in the prevalence of pre-diabetes. The higher prevalence of DM among men than women might be an accumulative effect of other risk factors such as smoking, tobacco use and alcohol consumption, which impact both TB and DM. The other reason could be the younger age of women than men since increasing age emerged as a significant risk factor for diabetes. In the current study, amongst those with DM, 9.3% were newly detected DM cases and 16% were known cases with DM.

Studies conducted in regions with dual burden had reported that the prevalence of DM ranged from 14–40% [19], [20]. A case control study conducted in Bangalore, South India, during 2001–2003 reported that chronic disease particularly diabetes was a significant risk factor for developing TB. The prevalence rates of diabetes in TB and non-TB subjects were 22.2% and 15.9% respectively [21]. Based on secondary analysis of countrywide data, another research group estimated that 18.4% of subjects with PTB also have DM in India [10]. A retrospective analysis of 2 years data on TB subjects from Saudi Arabia in 1998 revealed that 27% had DM [22]. Another study from Taiwan reported 16.9% of DM among TB patients [23]. All these reports indicate that routine screening for DM among TB patients should be encouraged in areas with high TB burden.

Alisjahbana et al. [19] reported prospective data from a cohort of patients with TB in Indonesia, where the prevalence of confirmed DM among patients with TB is 14.8% compared with 3.2% in general population. A nationwide INDIAB study [11] conducted in the general population of Tamil Nadu, South India, showed that the prevalence rates of diabetes and pre-diabetes were 10.4% and 8.3% respectively, substantially lower in comparison with the estimated prevalence of DM and pre-diabetes in the current study among TB patients conducted in the same period.

In the present study, among the TB patients identified with diabetes, more than 60% of them had been diagnosed with diabetes previously. It is known that long-term diabetes can impair the innate and adaptive immune responses necessary to counter the proliferation of TB [9]. This might be one of the reasons for the higher number of DM cases among the TB patients in the current study. In addition, the TB subjects also had the common risk factors associated with diabetes.

As in the general population age, BMI, positive family history of DM and sedentary occupation were the common risk factors associated with diabetes among TB patients, in the present study. The DM-associated risk factors among general population which were not seen in the current study were urban dwelling and income status. TB patients with DM were older compared to the non DM counterparts and while still underweight compared to the general population they had higher BMI relative to non DM TB patients. In the multinomial logistic regression analysis, odds ratio for age group >50 years was higher compared to those aged <30 years. There was a gradual increase in the odds ratio as the age increased in terms of five units. A sudden decrease in the odds ratio was observed in the age group 46–50 years, which could be attributed to the less number of patients in this age group. The TB patients with BMI 18.5–22.9 kg/m^2^ were double the time at risk of developing diabetes compared to the patients with BMI <18.5 kg/m^2^. The lesser distribution of TB patients in the overweight and obese categories, as per Asian Indian guidelines can be explained for the non-significant association with DM among these categories. Positive family history of diabetes was significantly associated with DM with an odds ratio of 3.08. Age, waist circumference, smoking habit and monthly income category 5000–10,000 INR emerged as independent risk factors significantly associated with pre-diabetes among TB patients. The highest odds ratio was shown for age category 41–45 years (6.96), waist circumference (OR 3.05), and smoking (OR 1.92) for pre-diabetes among TB patients.

Association of DM with PTB (OR 3.06, p<0.001) particularly for smear positive cases in this study was similar to that of the findings of Stevenson et al. [10] This higher association was not seen with extrapulmonary TB. The preponderance of development of DM among urban TB patients as reported by Stevenson et al. [10] was not noted in this study.

TB treatment outcome among TBDM patients is still not clear. Few studies reported that TB treatment outcome amongst DMTB subjects was good compared with non-DMTB subjects with the existing treatment [22], [24] whereas a study among Indonesian TB patients reported poor TB treatment outcome among DMTB subjects [19]. Besides other factors, the association between DM and TB can be attributed to poor glycemic control and lack of non-specific antibody production due to deficiency in innate and adaptive immune mechanism amongst subjects with diabetes. These were the facilitating factors for the resurgence of past infections and incidence of new TB cases among DM subjects. In our study, majority of known cases of DM (92.4%) were on regular treatment for diabetes and about 59% were treated in government health centres and the remaining were treated in private health care settings. Approximately 70% were treated with oral hypoglycaemic agents. About 34% of the patients had adequately controlled diabetes (HbA1c <7%). Despite treatment, the glycemic control was inadequate as more than 40% of DM subjects had HbA1c ≥9.0%. There was no significant difference in the proportion of relapsed, failure and treatment default cases between the various levels of glucose intolerance. No conclusions can be drawn on this as the present study was not designed to address this issue.

An earlier report stated that prevalence of diabetes might be overestimated in TB since TB can cause transient hyperglycaemia [25]. Thus, over-diagnosis might take place if tested for glucose prior to initiation of TB treatment. Majority of our study subjects were screened for DM by OGTT after 3 weeks of initiation of TB treatment. Ideally, glucose screening for DM diagnosis may be more appropriate after TB treatment has taken effect.

Several recent reports indicate the need to consider the increasing trend in prevalence of diabetes in countries like India, which will impact the TB burden as well [14], [26], [27]. Considering the growing trend in prevalence of diabetes and huge burden of latent TB infection amongst Indian population, it is necessary to focus on diagnosis of latent TB infection and screening for DM and ensuring good metabolic control amongst those diagnosed with DM. The role of possible chemoprophylaxis for subjects with DM and latent TB needs to be carefully considered and evaluated given the magnitude of the burden.

The study reports prevalence of DM among TB patients registered in the RNTCP. Since there is no active case detection under RNTCP, a community-based study would provide a better representative sample. This is one of the limitations of the present study.

Moreover, there is a need for greater collaboration between RNTCP and National Program for Prevention and Control for Cancer, Diabetes, Cardiovascular Diseases and Stroke (NPCDCS) in India to ensure that protocols and guidelines are in place to address the dual burden. Given the high association between TB and dysglycemia (half of TB cases with diabetes or pre-diabetes) and that more than a third of DM cases amongst people with TB were newly detected, a recommendation for universal screening for diabetes amongst people with TB would not seem out of place in a country like India with a high double burden. The limited resources and infrastructure for diabetes prevention and care at the primary setting make it even more necessary that the health resources and infrastructure for TB control should be further strengthened and serve the purpose of promoting prevention, early detection, and treatment of diabetes among TB patients particularly in India. Although such services can be offered for a limited time–during the course of TB treatment–the intensive contact through DOTS initiative may lay a strong foundation for lifelong good control through self-care training in diabetes while at the same time reduce risk of treatment failure, re-infection, and relapse.

In conclusion, this study revealed that about half of TB patients had either diabetes or pre-diabetes. Moreover, those with TBDM were more likely to have the infective form of TB. These findings pose a great challenge for TBDM control in India.
